# Measuring Psychological Trauma in the Workplace: Psychometric Properties of the Italian Version of the Psychological Injury Risk Indicator—A Cross-Sectional Study

**DOI:** 10.1155/2015/720193

**Published:** 2015-03-08

**Authors:** Nicola Magnavita, Sergio Garbarino, Peter C. Winwood

**Affiliations:** ^1^Department of Public Health, Università Cattolica del Sacro Cuore, 00168 Roma, Italy; ^2^Department of Neuroscience, Rehabilitation, Ophthalmology, Genetics and Maternal-Infantile Sciences (DINOGMI) and Department of Health Sciences, University of Genoa, 16126 Genoa, Italy; ^3^State Police Health Service Department, Ministry of the Interior, 00189 Roma, Italy; ^4^School of Psychology, Social Work, and Social Policy, University of South Australia, Adelaide, SA 5001, Australia

## Abstract

*Background*. The aim of this study was to cross-culturally adapt the Psychological Injury Risk Indicator (PIRI) and to validate its psychometric properties.* Methods*. Workers from 24 small companies were invited to self-complete the PIRI before undergoing their routine medical examination at the workplace. All participants (841 out of 845, 99.6%) were also asked to report occupational injuries and episodes of violence that had occurred at the workplace in the previous 12 months and were given the General Health Questionnaire (GHQ12) to complete.* Results*. Exploratory factor analysis revealed a 4-factor structure, “sleep problems,” “recovery failure,” “posttraumatic stress symptoms,” and “chronic fatigue,” which were the same subscales observed in the original version. The internal consistency was excellent (alpha = 0.932). ROC curve analysis revealed that the PIRI was much more efficient than GHQ12 in diagnosing workers who had suffered trauma (workplace violence or injury) in the previous year, as it revealed an area under the curve (AUC) of 0.679 (95% CI: 0.625–0.734) for the PIRI, while for the GHQ12 the AUC was 0.551 (not significant).* Conclusions*. This study, performed on a large population of workers, provides evidence of the validity of the Italian version of the PIRI.

## 1. Background

Work-related stress is a very important and complex phenomenon. Occupational health surveillance must check for the presence of environmental stressors in the workplace, determine how they are perceived by workers, and assess any possible resulting psychological damage. The occupational physician has a number of tools that can be used before regular medical examinations of workers to measure the risk factors, the perception of risk, and the ultimate psychological effects. The literature includes a large number of questionnaires that cover the first two areas, while there are relatively few screening tools to indicate psychological damage [1].

The Psychological Injury Risk Indicator (PIRI) is a recently developed instrument designed to identify the presence of psychological injury and to assess the level or degree of injury [2]. The authors conducted some pilot studies on police officers, as these first-responder workers are exposed to operational challenges in high-stress environments where they are sometimes subjected to direct physical threat and also to organizational demands and exhausting work schedules often encountered in paramilitary structured, male-dominated, hierarchical work groups [3]. Both traumatic operational events and threat/stressors of a more administrative nature, such as workplace discrimination, bullying, or internal discipline and control structures, may activate a complex and highly adaptive neural response within the brain—the threat response mechanism—which focuses physical and cognitive capacities on resolving the threat. When environmental circumstances cause it to be activated too frequently, and for excessively long periods, the abnormally high chemical stress of cells in the brain's limbic system (allostatic load) [4] results in a variety of characteristic behavioral, emotional, and capacity changes, the outcome of which may be uncertain [5, 6].

The PIRI pilot studies indicated that this instrument may assess the level or severity of psychological injury with good correspondence to a clinical assessment made by an experienced clinician. These studies indicated that further testing in larger groups, together with clinical assessment by multiple clinicians, was needed in order to confirm these findings and ascertain whether the PIRI is also a useful tool for the screening of workers.

In this study, the cross-cultural validity of the questionnaire was evaluated, and the Italian version of the PIRI was administered to workers from various sectors in which the risks were different from those experienced by police officers. The main purpose of this work was to verify the psychometric properties of the questionnaire. A second aim was to assess whether the PIRI could be useful in screening individuals who had suffered injury or violence at work.

## 2. Methods

### 2.1. Translation Procedure

The translation of the PIRI into Italian was carried out in accordance with well-accepted guidelines [7]. Firstly, an assessment of the linguistic accuracy of the PIRI was made by means of a standard “forward-backward” translation procedure. Independent translators and occupational physicians familiar with occupational psychology performed forward translation, while a native English speaker performed backward translation. Assessment of linguistic and conceptual equivalence was made during the translation process from English into Italian and vice versa. Sixteen subjects filled in the penultimate version and underwent a semistructured interview about problems in wording and answering. All the findings were reevaluated by the authors, although no further adjustment was required. The author of the original version of the questionnaire was available to clarify any doubts. The Italian version of the questionnaire that was finally adopted was therefore the result of corrections for any inconsistencies detected between the original version and the resulting draft. Finally, the questionnaire was administered to workers prior to their regular medical examination in the workplace. The PIRI was usually self-completed in 5–10 min, with a physician available to explain any questions that arose. On the whole, workers did not have difficulty in understanding the instructions or the items of the scale.

### 2.2. Population and Questionnaires

Workers from 24 small companies in the Latium region of Italy were invited to self-complete the PIRI and the General Health Questionnaire (GHQ12) [8] prior to their regular medical examination at the workplace. The GHQ12 is a well-known, validated, and frequently used self-report scale for assessing mental health. It was included to test the construct validity and responsiveness and to permit direct performance comparison with the PIRI scale in the screening of employees who had experienced trauma.

The PIRI included 30 questions. Questions 1–26 yielded 7 replies, with a score graded according to a Likert scale, comprising 4 subscales: A, “sleep problems” (6 items), B, “recovery failure” (5 items), C, “posttraumatic stress symptoms” (10 items), and D, “chronic fatigue” (5 items). Questions 27–30 were dichotomous (yes/no) items, corresponding to the Rapid Alcohol Problems Screen (RAPS 4) measure, a four-question quiz designed for detecting alcohol dependence [9]. The raw score total was standardized, so the range was between 0 and 100. The GHQ12 comprised 12 questions, each of which had 4 replies graded according to a Likert scale. The total score was standardized to make it homogeneous with the PIRI scale, so that the score ranged from 0 to 100.

Workers' participation was not mandatory; however, almost all the workers agreed to fill in the questionnaires. Written informed consent for participation in the study was obtained from participants. All participants (841 out of 845, 99.6%) were also invited to report occupational injuries and episodes of violence that had occurred in the workplace in the previous 12 months.

The final sample included 841 workers, 277 (32.9%) males and 564 (67.1) females, with a mean age of 42.8 ± 10.6 years. They were employed in stores and supermarkets, social and home care, education, fuel distribution, construction, and offices.

Before the survey we assessed the degree to which the results of PIRI are consistent over time administering the questionnaire to 12 volunteers twice a week away.

This study was approved by the Ethics Committee of the Università Cattolica del Sacro Cuore of Roma, and permission was obtained from the author of the original version.

### 2.3. Statistical Analyses

The internal consistency of the PIRI was evaluated using Cronbach's alpha. The item-total correlation and changes in alpha due to deleted items were examined in order to determine how each item influenced the reliability of the tool. Only items that had a significant correlation with the total score and produced a reduction in alpha value if the item was removed were studied in subsequent analyses.

The stability of the questionnaire was assessed by giving the PIRI to the same respondents on two separate occasions using the test-retest intraclass correlation coefficient (ICC) [10]. Anova *F* test was used to analyze the difference between trials. ICC was calculated using two-way mixed model, type consistency, averaged measures.

Construct validity, that is, the ability to measure the underlying concept of interest in occupational medicine, was assessed by comparing the PIRI with GHQ12 using Spearman's rho correlation coefficient.

Exploratory factor analysis (EFA) [11, 12] was conducted to test the dimensionality of the scale, to identify interrelationships among group items that formed unified concepts, and to determine whether the Italian version maintained the same structure as the original Australian form that is classically divided into 5 subscales. EFA was performed after considering whether Bartlett's test of sphericity, assessing if the correlation matrix of data is an identity matrix with all items unrelated, was significant and the Kaiser-Meyer-Olkin (KMO) criterion, which tests the sampling adequacy to ensure that the scale items are relevant for factorial analysis, was ≥0.80. The Kaiser rule was used to determine the number of extracting factors (eigenvalues >1). As this method (i.e., the default method in many statistical package programs) is not recommended when used as the sole cut-off criterion as it may overinflate the number of factors [13], we also repeated the EFA, dropping one factor. Varimax rotation of factors with the Kaiser optimization technique was used to make the output more understandable. Interpretation of the factors was based on observation of factor loadings. Items with a loading of over 0.50 in one factor, and less than 0.30 in each of the remaining factors, were interpreted to be indicative of that factor.

Responsiveness, that is, the extent to which changes in questionnaire measure relate to external changes, was studied by Receiver Operating Characteristics (ROC) curve analysis. A ROC analysis was calculated on the PIRI and GHQ12 score using the dichotomous “trauma experience” (injury or workplace violence in the previous year) as the external criterion. ROC curves were utilized to identify the most discriminant PIRI total score cut-off (i.e., the one that was able to distinguish workers who had suffered psychological trauma) from all others. Using the ROC curve, the diagnostic efficacy of each questionnaire was described in terms of sensitivity (probability that the measure correctly classified workers who had experienced trauma) and specificity (probability that the measure correctly classified workers who had not experienced trauma). Values for sensitivity and for false-positive rates (1 − specificity) were plotted on the *y*- and the *x*-axis of the curve and the area under the curve (AUC) represented the probability that the measure correctly classified workers as positive or negative with respect to trauma. This area theoretically ranges from 0.5 (inaccuracy in discriminating) to 1.0 (perfect accuracy) and an AUC around 0.70 is considered to be acceptable [14].

Statistical analyses were conducted with the IBM/SPSS package (version 20.0).

## 3. Results

### 3.1. Descriptive Analyses

The survey results are summarized in Table 1.

The workers showed willingness to take part in the survey and on the whole encountered no difficulty in completing the PIRI. Workers were able to seek the advice of a physician if they had any doubts. The most frequent queries involved questions 27–30, relating to the maladaptive use of alcohol. All the workers in this study had been the target of an information campaign on the use of alcohol and actions against drinking on the job since 2008, so alcohol abuse was virtually nonexistent in this cohort. Consequently, the answers to these questions were almost all negative, making it impossible to analyze this part of the questionnaire.

Some workers who had not suffered any recent trauma observed that the questionnaire contained questions about “particularly upsetting or distressing events or experiences you have encountered during the course of your work” but that nothing had actually happened to them.

### 3.2. Internal Consistency and Reliability

Test-retest reliability measured on 12 subjects in two separate tests with an interval of one week gave no difference between trials and an intraclass correlation coefficient ICC = 0.931, indicating a high degree of reliability.

The PIRI scale revealed a high level of internal consistency. Cronbach's alpha value computed on the whole questionnaire was 0.927; each of the first 26 items had a substantive correlation with the total and provided a relevant contribution to the scale because alpha decreased when each item in turn was deleted.

In our sample alcohol-related problems were virtually absent. The last four items (from 27 to 30), concerning alcohol use, had 3359 negative and only 5 positive answers. Consequently, items were not correlated with the total and, if removed, caused an increase in the consistency of the scale. If questions about alcohol use were excluded, Cronbach's alpha value for the first 26 items of PIRI rose to 0.932.

Internal consistency ranged from good to excellent for each of the subscales: for part A, “sleep problems” (6 items), alpha = 0.765; part B, “recovery failure” (5 items), alpha = 0.919; part C, “posttraumatic stress symptoms” (10 items), alpha = 0.913; and part D, “chronic fatigue” (5 items), alpha = 0.776. Part E of the questionnaire, containing 4 binary items on maladaptive alcohol use, could not be evaluated due to the fact that, out of the 841 workers, only 4 gave at least one affirmative response. For this reason, items from 27 to 30 of the PIRI were excluded from subsequent analyses.

The GHQ12 had an excellent internal consistency (alpha = 0.830).

### 3.3. Construct Validity

A significant correlation was found between the total scores of the PIRI and the GHQ12 (Spearman's rho = 0.415, *P* < 0.0001).

### 3.4. Structural Validity

A KMO coefficient of 0.937 and statistical significance (*P* < 0.001) of Bartlett's test for sphericity enabled us to perform factor analysis on items 1–26 of the PIRI. Five factors had an eigenvalue higher than 1 and the first explained 38.2% of the variance, while all factors together explained 62.5%.  Table 2 shows the item-factor loadings using Varimax rotation and the single item communalities. Item communalities followed the same distribution as the original version of the questionnaire, with the only exception of part C “posttraumatic stress symptoms” which was split into two factors: remind symptoms (3 items) and psychosocial symptoms (7 items). When we forced the analysis to elicit only 4 factors, these two factors collapsed into a single factor, which explained 38.3% of variance. The overall explained variance with a 4-factor solution was 58.5% (Table 3).

### 3.5. External Responsiveness

**Figure 1:**
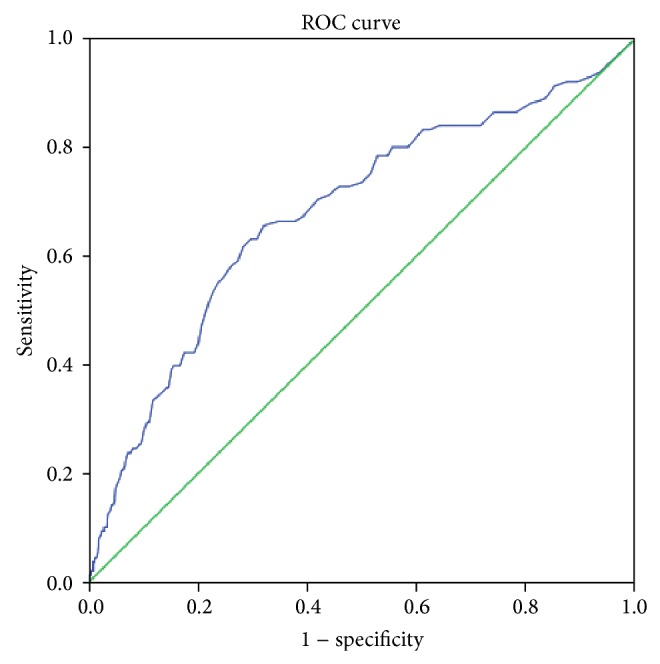
ROC curve for PIRI, workers with trauma.

The ROC analysis carried out on the PIRI standardized score (calculated on 26 items) revealed an AUC of 0.68 (95% CI 0.63–0.73), thus showing acceptable discriminative capacity; the cut-off point that best discriminated between workers with and without previous trauma was 19.5 (sensitivity 66%, specificity 68%) (Figure 1). Conversely, the GHQ12 score had a very low and nonsignificant AUC of 0.55 (95% CI 0.49–0.61).

## 4. Discussion and Conclusions

The present study provides evidence of the validity of the Italian version of the PIRI. We translated the questionnaire into Italian, adapted it to the original English version, and then tested it on a large sample of workers from various occupational areas. Our results demonstrate high internal consistency, construct and structural validity, and responsiveness of the instrument.

The internal consistency of the PIRI was satisfactory as Cronbach's alpha was above 0.90 and each of the first 26 items had a substantive correlation with the total. Furthermore, Cronbach's alpha decreased when each item was deleted, confirming the relevant contribution of each item to the scale. A poor correlation was found only between questions concerning alcohol use and the overall score and, when these were deleted, the alpha increased.

The structural validity of the Italian version of the PIRI was exactly the same as the Australian version. Subscales, A, “sleep problems” (6 items), B, “recovery failure” (5 items), C, “posttraumatic stress symptoms” (10 items), and D, “chronic fatigue” (5 items), in the Italian version are corresponding to the original.

The PIRI is proved to be strictly related to the GHQ12 and, therefore, to have the same construct validity as the latter which is currently the most common assessment of mental wellbeing [15]. This screening test, developed to detect people at risk of developing psychiatric disorders, is a measure of the common mental problems of depression, anxiety, somatic symptoms, and social withdrawal [15]. Two factors, expressing anxiety/depression and social dysfunction, proved to be highly correlated with each other and sufficiently stable in cross-cultural comparisons [16]. However, the PIRI seems more effective than the GHQ in determining damage resulting from traumatic stress. When these two instruments were compared in workers who had suffered a trauma such as an injury or physical or verbal aggression, while the GHQ failed to show any diagnostic power, the PIRI proved to be a test of acceptable sensitivity and specificity. In this context, it should be noted that the method used to conduct the survey limited the efficacy of the test. In fact, since the workers were subjected to annual medical examination, the time interval within which the adverse event could have happened was 12 months, while the PIRI referred to events that had occurred in the previous three months.

The average score of the PIRI in our cohort is significantly lower and the number of cases that may be considered indicative of psychological injury is significantly lower than that observed in studies of Australian police and patients with posttraumatic stress disorder PTSD [2, 3]. Our observation in fact was conducted on “healthy,” not first-line, workers. The effectiveness of the PIRI also in this cohort shows that this questionnaire can have a much wider application than the initial one; for example, it can be used to measure psychological discomfort during common jobs and outside major stressful events. The theoretical basis, development, content and construct validity, and reliability of the original English version of the PIRI suggest that it has the potential to be a useful assessment of psychological injury in workers who have experienced occupational trauma of moderate intensity, such as a work accident or workplace harassment.

The PIRI questionnaire contains two subscales, “recovery failure” and “chronic fatigue,” making it a potentially useful tool in studies of presenteeism, sickness absence, and job performance. In future studies it would be interesting to compare the PIRI with other outcome measures such as the Health and Work Performance Questionnaire (HPQ) [17], the Work Limitations Questionnaire (WLQ) [18], and the Nurses Work Functioning Questionnaire [19].

A limitation of our study is that it was conducted on a cohort of workers who had already been screened for alcohol; consequently, the frequency of maladaptive behavior related to alcohol was lower than expected. Longitudinal studies conducted in populations of different employment sectors can help to improve the knowledge of this tool.

This study therefore demonstrates the validity of its Italian version, providing a potentially useful instrument for assessing a distinct cluster of symptoms that have been found to predict long-term dysfunctions.

## Figures and Tables

**Table 1:** Population and data.

	Cases, *N*	%
Male	277	32.9
Female	564	67.1
Physical assault	37	4.4
Threat	30	3.6
Harassment	70	8.3
Injury	33	3.9
Any trauma	125	14.9

	Mean	s.d.

Age, years	42.8	10.6
PIRI (range 0–100)	18.53	15.90
GHQ12 (range 0–100)	18.59	8.57

**Table 2:** Rotated matrix of components, 5-factor solution. Items correlated with the specific factor are evidenced in bold.

	Factor				
	1	2	3	4	5
PIRI1	−.012	.109	.110	**.703**	.215
PIRI2	.105	.097	.194	**.625**	.187
PIRI3	.214	.166	.104	**.537**	−.080
PIRI4	.193	.309	.352	**.453**	.210
PIRI5	.238	.284	.225	**.556**	.138
PIRI6	.247	.233	.004	**.683**	.046
PIRI7	.066	**.777**	.169	.180	.121
PIRI8	.083	**.843**	.180	.162	.125
PIRI9	.166	**.853**	.144	.169	.076
PIRI10	.184	**.801**	.149	.210	.113
PIRI11	.220	**.772**	.231	.213	.139
PIRI12	.293	.150	.134	.138	**.789**
PIRI13	.239	.155	.133	.194	**.802**
PIRI14	.381	.134	.100	.119	**.650**
PIRI15	**.727**	.104	.174	.198	.096
PIRI16	**.784**	.125	.186	.167	.125
PIRI17	**.768**	.165	.163	.177	.164
PIRI18	**.560**	.157	.257	.099	.451
PIRI19	**.650**	.201	.242	.144	.357
PIRI20	**.639**	.158	.268	.069	.371
PIRI21	**.583**	.085	.256	.204	.296
PIRI22	.093	.144	**.586**	.160	.243
PIRI23	.288	.098	**.651**	.172	.138
PIRI24	.211	.204	**.708**	.167	.074
PIRI25	.198	.198	**.647**	.185	−.032
PIRI26	.202	.159	**.713**	.003	.105

**Table 3:** Rotated matrix of components, 4-factor solution. Items correlated with the specific factor are evidenced in bold.

	Factor			
	1	2	3	4
PIRI1	.128	.121	.053	**.709**
PIRI2	.196	.101	.163	**.630**
PIRI3	.096	.143	.165	**.536**
PIRI4	.278	.312	.330	**.461**
PIRI5	.262	.276	.236	**.561**
PIRI6	.204	.213	.049	**.684**
PIRI7	.129	**.779**	.157	.188
PIRI8	.145	**.844**	.171	.171
PIRI9	.173	**.845**	.166	.176
PIRI10	.210	**.794**	.165	.217
PIRI11	.254	**.766**	.245	.222
PIRI12	**.749**	.186	.010	.155
PIRI13	**.717**	.195	−.006	.212
PIRI14	**.719**	.155	.029	.133
PIRI15	**.597**	.064	.302	.200
PIRI16	**.658**	.084	.319	.169
PIRI17	**.673**	.127	.285	.180
PIRI18	**.715**	.154	.263	.110
PIRI19	**.718**	.185	.290	.152
PIRI20	**.720**	.146	.308	.078
PIRI21	**.626**	.070	.302	.211
PIRI22	.230	.164	**.521**	.170
PIRI23	.302	.098	**.649**	.180
PIRI24	.202	.206	**.703**	.175
PIRI25	.120	.191	**.666**	.190
PIRI26	.218	.164	**.698**	.011

% Variance	20.947	14.867	12.016	10.679

Construct	PTSD symptoms	Recovery failure	Chronic fatigue	Sleep problems

## Abbreviations

PIRI: Psychological Injury Risk Indicator
GHQ12: General Health Questionnaire
EFA: Exploratory factor analysis
ROC: Receiver Operating Characteristic curve
AUC: Area under the curve
KMO: Kaiser-Meyer-Olkin criterion
IBM/SPSS: International Business Machines Corporation/Statistical Package for Social Sciences
PTSD: Posttraumatic stress disorder.
